# A general design of caging-group-free photoactivatable fluorophores for live-cell nanoscopy

**DOI:** 10.1038/s41557-022-00995-0

**Published:** 2022-07-21

**Authors:** Richard Lincoln, Mariano L. Bossi, Michael Remmel, Elisa D’Este, Alexey N. Butkevich, Stefan W. Hell

**Affiliations:** 1 Department of Optical Nanoscopy, Max Planck Institute for Medical Research, Heidelberg, Germany; 2 Department of NanoBiophotonics, Max Planck Institute for Multidisciplinary Sciences, Göttingen, Germany; 3 Optical Microscopy Facility, Max Planck Institute for Medical Research, Heidelberg, Germany

**Keywords:** Fluorescence spectroscopy, Single-molecule fluorescence, Fluorescence imaging, Super-resolution microscopy, Fluorescent dyes

## Abstract

The controlled switching of fluorophores between non-fluorescent and fluorescent states is central to every super-resolution fluorescence microscopy (nanoscopy) technique, and the exploration of radically new switching mechanisms remains critical to boosting the performance of established, as well as emerging super-resolution methods. Photoactivatable dyes offer substantial improvements to many of these techniques, but often rely on photolabile protecting groups that limit their applications. Here we describe a general method to transform 3,6-diaminoxanthones into caging-group-free photoactivatable fluorophores. These photoactivatable xanthones (PaX) assemble rapidly and cleanly into highly fluorescent, photo- and chemically stable pyronine dyes upon irradiation with light. The strategy is extendable to carbon- and silicon-bridged xanthone analogues, yielding a family of photoactivatable labels spanning much of the visible spectrum. Our results demonstrate the versatility and utility of PaX dyes in fixed and live-cell labelling for conventional microscopy, as well as the coordinate-stochastic and deterministic nanoscopies STED, PALM and MINFLUX.

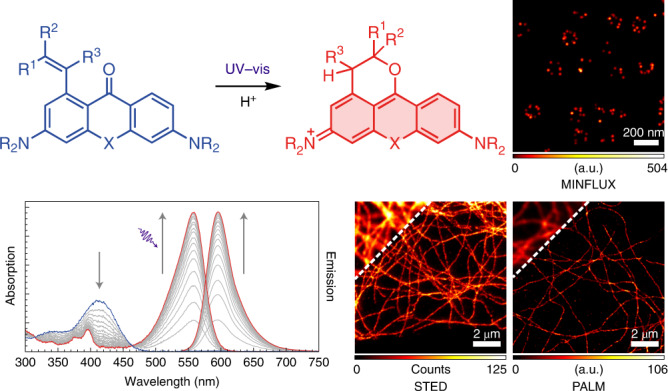

## Abstract (web summary)

The design of photoactivatable fluorophores—which are required for some super-resolution fluorescence microscopy methods—usually relies on light-sensitive protecting groups imparting lipophilicity and generating reactive by-products. Now, it has been shown that by exploiting a unique intramolecular photocyclization, bright and highly photostable fluorophores can be rapidly generated in situ from appropriately substituted 1-alkenyl-3,6-diaminoxanthone precursors.

## Main

Fluorescence nanoscopy has revolutionized our ability to visualize (living) cells by extending the limits of optical imaging to single-digit nanometre resolution, and by enabling minimally invasive observation of the internal nanoscale structures and dynamics of biological samples with molecular specificity^1–4^. Central to these techniques are chemically specific fluorescent labels and the intrinsic control between fluorescent (on) and non-fluorescent (off) states of the fluorophores. This sequential off–on transition is key to separating adjacent fluorophores at molecule-scale proximities. Photoactivatable or caged dyes—in which the off–on transition is irreversible and triggered by light—render these nanoscopy techniques very powerful, because they eliminate the need for specific imaging buffers and high intensities of UV light. Such requirements are prevalent in single-molecule-based microscopy, such as photo-activated localization microscopy (PALM) or stochastic optical reconstruction microscopy (STORM), to drive commonly used fluorophores (for example, cyanines) between non-fluorescent and fluorescent states^5–7^, as well as enabling high-density single-particle tracking^8–10^. Most recently, photoactivatable dyes have been used to reduce the fluorescence background in DNA-PAINT^11^ and to increase the number of cellular structures that may be simultaneously imaged in stimulated emission depletion (STED) microscopy through channel duplexing^12^.

Rhodamine dyes have emerged as some of the most widely employed fluorophores in fluorescence microscopy and nanoscopy due to the remarkable tunability of their optical and chemical properties^13–15^, cell membrane permeability^16^, photostability^17^ and brightness^18^. In particular, silicon rhodamines^19^ are often favoured for their intrinsic redshifted emission, fluorogenic behaviour^20^ and live-cell compatibility^21–23^. However, the reported caging strategies for rhodamines rely on ‘locking’ the dyes in a non-fluorescent form, either through installation of photolabile protecting groups on the nitrogen atoms (such as with nitroveratryloxycarbonyl^7,24,25^ or nitroso^26^ groups) or by synthetic transformation of the lactone ring into the corresponding cyclic α-diazoketones^9,27^. The former strategy restricts the attainable substitution patterns, reduces water solubility and yields stoichiometric amounts of potentially toxic by-products upon photoactivation. The latter strategy, meanwhile, suffers from varying uncaging efficiencies and the concomitant formation of non-fluorescent side products, whose abundance depends on the medium and substitution pattern^27,28^.

Accordingly, caging-group-free, compact photoactivatable and biocompatible fluorophores are highly desirable in fluorescence microscopy and nanoscopy applications, enabling lower-molecular-weight labels, provided that the photoactivation is rapid, complete and free of by-products. Recently, the photoactivation of a Si-pyronine analogue was demonstrated, where the fluorophore was initially masked with an exocyclic double bond at the 9-position of the Si-xanthene scaffold^29^. Upon UV irradiation in aqueous solution, protonation of the exocyclic double bond yielded the fluorescent 9-alkyl-Si-pyronine. The resulting cationic fluorophore, however, was susceptible to formation of non-fluorescent nucleophilic addition products with thiols and water, limiting its applicability.

Inspired by the long-established radical photochemistry of benzophenone and other diarylketones, we have now designed, and report herein, a class of functionalized xanthones, which, upon one- or two-photon excitation, convert efficiently and cleanly into the corresponding dihydropyran-fused pyronine dyes. These photoactivatable xanthone (PaX) dyes can be prepared from readily available starting materials via a straightforward and efficient three-step synthetic route, also compatible with carbon- and silicon-bridged analogues, to yield a family of fluorophores spanning much of the visible spectrum. In particular, PaX-derived Si-pyronine dyes display good live-cell compatibility, resilience to nucleophiles, and an unprecedented photostability for orange-emitting (TAMRA-like) fluorophores. We highlight the utility of PaX dyes and labels in optical microscopy and nanoscopy techniques, in fixed and living cells, including STED, photo-activated localization microscopy (PALM) and minimal photon fluxes (MINFLUX).

## Results and discussion

### Synthetic design and proposed mechanism of photoactivation

**Fig. 1:**
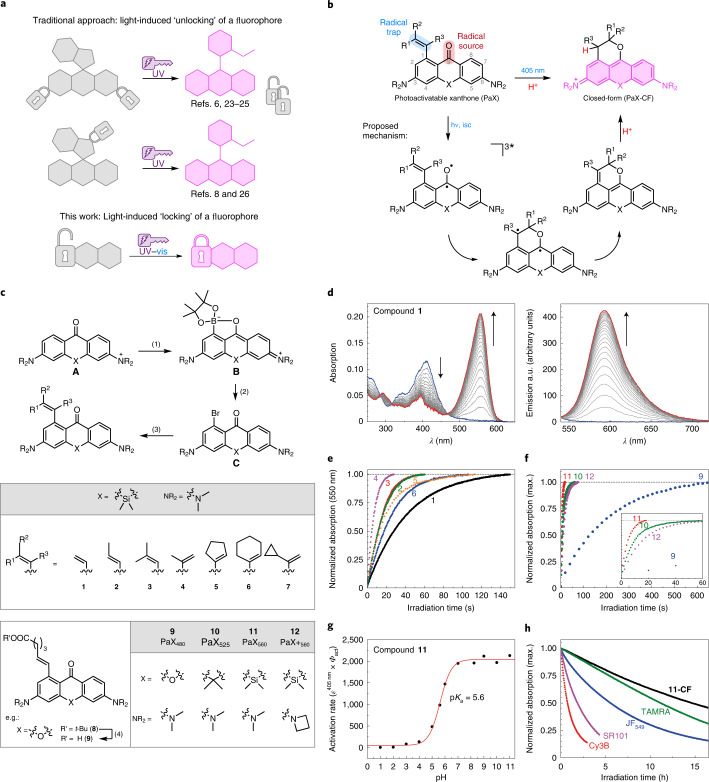
Design, synthesis and characterization of PaX dyes. **a**, Whereas traditional strategies for photoactivatable dyes for nanoscopy rely on the release (‘unlocking’) of caging groups, our approach relies on the light-induced assembly (‘locking’) of a fluorophore. **b**, General structure of a PaX with a 1-alkenyl radical trap and its 9-alkoxypyronine photoproduct (closed-form, **CF**), and the proposed photoactivation mechanism. **c**, Synthetic route for the preparation of PaX. (1) B_2_pin_2_, [Ir(cod)(OMe)]_2_, AsPh_3_, *n*-octane, 120 °C, 22 h; (2) CuBr_2_, KF, pyridine, DMSO/H_2_O, 80 °C, 30 min; (3) RB(OH)_2_, RBpin or RBF_3_K (R = alkenyl), Pd(dppf)Cl_2_, K_2_CO_3_, dioxane/H_2_O, 80 °C, 3–18 h; (4) CH_2_Cl_2_/TFA 3:1, r.t., 1 h. **d**, Temporal evolution of the absorption and fluorescence spectra of **1** (1.66 µg ml^−1^) irradiated in phosphate buffer (100 mM, pH 7; *λ*_act_ = 405 nm). **e**, Comparative photoactivation kinetics of Si-bridged PaX **1**–**6**, under the same conditions as in **d**. **f**, Comparative photoactivation kinetics of PaX dyes **9**–**12**, under the same conditions as in **d**. Inset: magnified view of the 0–60 s time region. **g**, Comparative photoactivation kinetics of **11** (3.8 µM) in phosphate buffer (100 mM) at different pH values (*λ*_act_ = 405 nm). **h**, Photo-fatigue resistance of **11-CF** and established commercial fluorophores, with similar spectral properties, measured in phosphate buffer (*λ*_exc_ = 530 nm). Source data

In our search for minimalistic photoactivatable fluorophores, we reasoned that the concept of employing photochemical reactions to assemble or ‘lock’ fluorophores, rather than ‘unlocking’ photocleavable caging elements, would provide an improved alternative to caged rhodamine dyes (Fig. 1a)—a strategy similar to photochromic diarylethenes^30^. Diarylketones are known photoinitiators of radical reactions^31^ due to their high inherent rate of intersystem crossing (via spin–orbit coupling) and their triplet states with diradical character^32,33^. We hypothesized that their photochemistry would be extendable to 3,6-diaminoxanthones, which are utilized as precursors in the synthesis of rhodamines^34–36^. With the introduction of a suitable intramolecular radical trap onto the xanthone scaffold, a juxtaposition of a radical source (diaryl ketone) and a radical trap (styrene) could be exploited to photoassemble 9-alkoxypyronine fluorophores through a light-triggered cascade (Fig. 1b)^37^.

To investigate the effects of substitution of the radical acceptor, we first synthesized a series of photoactivatable Si-xanthones (**1**–**7**; Fig. 1c). The target compounds were prepared by an Ir-catalysed, chelation-assisted, *ortho*-selective C–H borylation of the diaryl ketone (**A**)^38^. Conversion of the resulting boronate ester (**B**) into the corresponding aryl bromide (**C**) was carried out with a CuBr_2_–pyridine system^39^ in the presence of KF (details are provided in the Supplementary Information). A series of alkene substituents were then installed using standard Suzuki–Miyaura cross-coupling reaction conditions. Compounds **1**–**6** showed a strong absorption band (*ε* ≈ 10^4^ M^−1^ cm^−1^) at ~400 nm, characteristic of Michler’s ketone and its analogues (Supplementary Table 1 presents the photophysical characterization). Upon irradiation in protic media (for example, phosphate buffer, 100 mM, pH 7), compounds **1**–**6** underwent rapid and complete conversion to give highly fluorescent ‘closed-form’ (CF) products with TAMRA-like spectral properties (**1-CF** to **6-CF**; Fig. 1d and Supplementary Fig. 1). Liquid chromatography mass spectrometry (LC-MS) analysis of the reaction mixtures revealed no by-products for most samples. The measured quantum yields of photoactivation (*Φ*_PA_) ranged from 1 × 10^−2^ to 6 × 10^−2^ (Supplementary Table 1). The rate of photoactivation was slowest for vinyl-substituted compound **1**, increased with additional substitution of the alkene, and was highest for compound **4**, possibly due to a favourable orientation of the alkene induced by the α-methyl substituent (Fig. 1e). To confirm the formation of predicted 9-alkoxypyronine product **1-CF**, a solution of compound **1** in methanol was irradiated with a 405-nm light-emitting diode (LED) in a batch photoreactor (see Supplementary Information for details), and the resulting product was isolated and fully characterized by NMR and high-resolution mass spectrometry (HR-MS) analysis (Supplementary Fig. 2), confirming the expected dihydropyran ring fusion. A solvent-dependent protonation step was confirmed by conducting photolysis in methanol-*d*_4_ (resulting in deuterium incorporation at the benzylic position) and by the absence of efficient photoactivation in aprotic solvents such as 1,4-dioxane. Deoxygenating the solvent increased the rate of photoactivation, confirming the role of the xanthone triplet state. Photolysis of **1** (4.8 µM) in the presence of millimolar concentrations of the radical trap 4-hydroxy-TEMPO resulted in the formation of a PaX-TEMPO adduct (Supplementary Fig. 3); however, the radical clock probe **7** showed no evidence of cyclopropane ring-opening upon photoactivation (Supplementary Fig. 4).

To render the PaX dyes suitable for bioconjugation, xanthone **9** (PaX_480_), anthrone **10** (PaX_525_) and Si-xanthone **11** (PaX_560_), along with its bis-azetidine analogue **12** (PaX+_560_), were prepared (Fig. 1c) bearing alkenyl substituents with a short carboxylate-terminated spacer. The keto forms of **9**, **10** and **11** showed initial absorbance maxima at 399, 408 and 414 nm, respectively (Supplementary Fig. 5 and Supplementary Table 2). The rate of photoactivation yielding the pyronine dyes (with absorption/emission maxima at 480/514 nm for **9-CF**, 524/564 nm for **10-CF** and 558/596 nm for **11-CF**) decreased in the order **11** > **10** > **9** (Fig. 1f), without noticeable by-product formation by LC-MS analysis, and the closed forms remained stable for at least 1 h at pH 7 (Supplementary Fig 6). As we expected, the azetidine auxochromic groups had little impact on the spectral properties of both the Si-xanthone (**12**) and Si-pyronine (**12-CF**) forms, but instead reduced the rate of photoactivation compared to the bis(*N*,*N*-dimethylamino) analogue (**11**). Fluorophore **12-CF** demonstrated remarkably improved emission quantum efficiency (0.92 versus 0.48 for **11-CF**), which can be attributed to the suppression of transfer into a twisted internal charge transfer state upon excitation^18^.

Screening the photoactivation properties of **11** over a range of biologically relevant pH values (Fig. 1g and Supplementary Fig. 7a) revealed a six-fold decrease in the photoactivation rate in acidic media (pH 4.3) as compared to neutral, and only small rate changes at basic pH values (up to 9.0). The low pH-dependence of the activation rate is similar to previous observations on the protonation of the benzophenone triplet excited state^40,41^, supporting the assumed involvement of this diradical in the activation mechanism. At high pH values, slow hydrolysis of **11-CF** was observed (Supplementary Fig. 7b); however, there was no difference in the absorption and emission spectra of **11-CF** and no change in product composition was detected by LC-MS up to pH 8.5 (Supplementary Fig. 7c), indicating little observable pH-sensitivity for this dye across the biologically relevant pH range. Furthermore, photoactivation of **11** proceeded cleanly in buffered solutions (pH 7) containing 2 mM mercaptoethylamine or glutathione (Supplementary Fig. 8), anticipating a lack of unwanted radical or electrophilic reactivity towards biomolecules, and a potential orthogonality with the single-molecule localization microscopy (SMLM) blinking buffers used for cyanine dyes^5^. Finally, we assessed the photostability of **11-CF**, benchmarking it against a series of commercially available dyes with similar spectral properties (Fig. 1h), and found that **11-CF** outperformed all of the tested fluorophores (for details, see Supplementary Information and Supplementary Figs. 9 and 10).

### Caging-group-free photoactivatable labels for nanoscopy

**Fig. 2:**
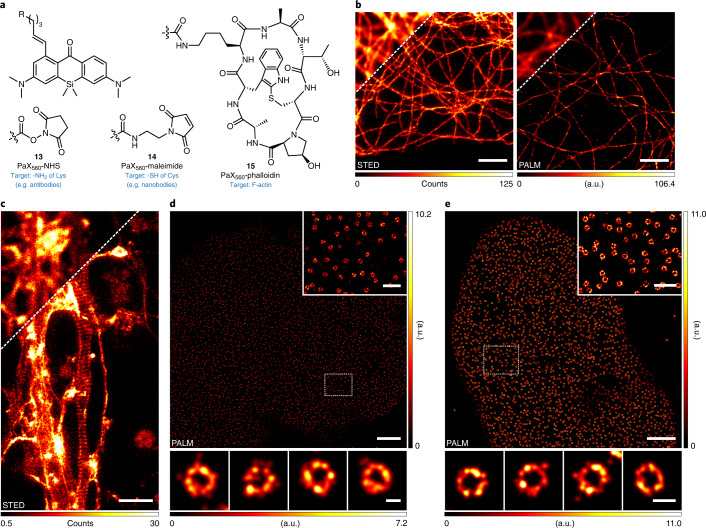
Photoactivatable labels for optical nanoscopy. **a**, Structures of PaX_560_ derivatives for bioconjugation (**13**, **14**) and actin labelling (**15**). **b**, STED (left) and PALM (right) images of microtubules in COS-7 cells labelled by indirect immunofluorescence with a secondary antibody bearing **13**. Preactivation to **13-CF** for STED imaging was achieved with widefield illumination (AHF analysentechnik AG, 4,6-diamidino-2-phenylindole filter set F46-816). **c**, Actin structures of the periodic membrane cytoskeleton in the axon of fixed primary hippocampal neuron cultures labelled with **15** and mounted in Mowiol. Preactivation to **15-CF** for STED imaging was achieved with widefield illumination (AHF, enhanced green fluorescent protein (EGFP) filter set F46-002) followed by a 518-nm laser. Image data were smoothed with a 1-pixel low-pass Gaussian filter. **d**, PALM image of NPCs in COS-7 cells labelled via indirect immunofluorescence with an anti-NUP98 primary antibody and a secondary nanobody labelled with **14**. Inset: magnified view of the region marked in the overview image. Bottom row: individual NPCs. **e**, PALM image of NPCs in HeLa-Kyoto cells expressing NUP107-mEGFP labelled with anti-GFP nanobodies conjugated to **14**. Inset: magnified view of the region marked in the overview image. Bottom row: individual NPCs. Scale bars: 2 μm (**b**–**e**, main images), 500 nm (**d**,**e** insets), 50 nm (**d**, bottom row), 100 nm (**e**, bottom row).

Encouraged by the versatility of the PaX mechanism, we proceeded to construct targeted labels for fluorescence microscopy and nanoscopy. For indirect immunolabelling (with secondary antibodies or nanobodies), an amino-reactive *N*-hydroxysuccinimide (NHS) ester (**13**) and a thiol-reactive maleimide (**14**) derivative of PaX_560_ were prepared (Fig. 2a), along with the NHS esters of PaX_480_, PaX_525_ and PaX+_560_ (Supplementary Figs. 11a and 16–18). For actin labelling in fixed cells, a phalloidin derivative (**15**) of PaX_560_ was assembled (Fig. 2a).

Thanks to their remarkable photo-fatigue resistance, we reasoned that PaX dyes would be strong candidates for STED imaging. We tested their performance by indirect immunofluorescence labelling of microtubules in fixed COS-7 cells. The fluorescent form of the dye was generated in situ (405-nm photoactivation) before STED imaging with 561-nm and 660-nm light for excitation and STED, respectively. Super-resolved images of microtubules were successfully acquired for antibody conjugates of PaX_560_ (**13**; Fig. 2b), as well as of PaX_525_ and PaX+_560_ (**17**,**18**; Supplementary Fig. 11b), demonstrating their compatibility with STED nanoscopy. The specificity of PaX_560_-phalloidin (**15**) for actin was validated in fixed neuron cultures in which the periodic membrane cytoskeleton structure of the axon was visualized by STED (Fig. 2c).

We next tested the performance of our photoactivatable labels in SMLM^42,43^. With this aim, PALM imaging was carried out on indirectly immunolabelled microtubules, and super-resolved images could be obtained for antibody conjugates bearing **13** (Fig. 2b) and **16**–**18** (Supplementary Fig. 11c). Thanks to the efficient photoactivation mechanism, very low powers (<100 µW) of activation light were required. Importantly, all samples were imaged in phosphate buffered saline (PBS) or in Mowiol, without the need for special blinking buffers or photostabilizing agents.

To further benchmark the utility of PaX labels for PALM imaging, indirect immunofluorescent labelling of nuclear pore complexes (NPCs) was conducted with a primary anti-NUP-98 antibody and secondary anti-rabbit nanobodies bearing **14** (Supplementary Fig. 12a,b). The PALM images of NPCs (Fig. 2d) were comparable in quality to those acquired through more demanding methods (for example, qPAINT^44^). Alternatively, the large-sized (~150 kDa) primary antibodies could be avoided to improve labelling precision^45^ in cell lines expressing an mEGFP^46^ (~27 kDa) fusion to NUP107 when combined with anti-GFP nanobodies labelled with **14** (Fig. 2e).

### Targeted labels for live-cell imaging

**Fig. 3:**
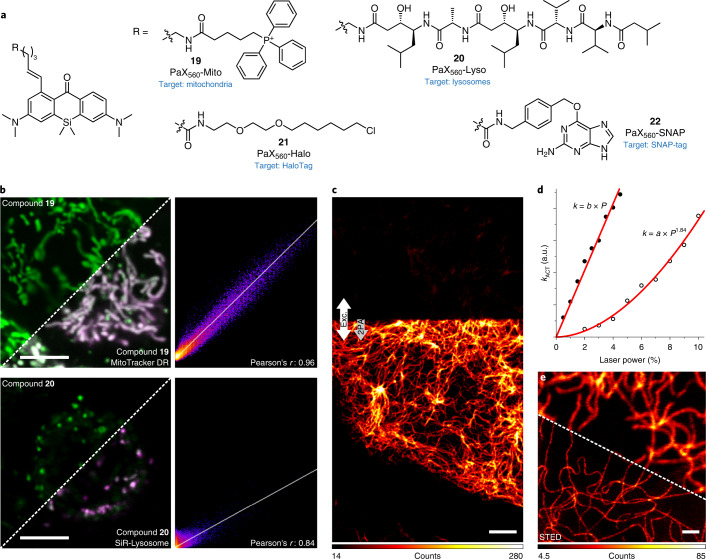
Imaging with photoactivatable PaX labels in living cells. **a**, Structures of PaX_560_ derivatives (**19**–**22**) for live-cell imaging. **b**, Confocal images and corresponding Pearson correlation analysis of COS-7 cells co-incubated with **19** (200 nM) and MitoTracker Deep Red (50 nM, top row) or **20** (20 nM) and SiR-lysosome (200 nM, bottom row). Conversion to **19-CF** and **20-CF** was achieved with a 355-nm laser. **c**, Confocal image of vimentin filaments labelled with **21** (200 nM) in U2OS cells before activation (upper portion) and with two-photon activation (2PA) (lower portion, indicated by the arrows). **d**, Plot of activation rate versus laser power for a one-photon (355 nm, 0.3 µW at 100%) or two-photon activation laser (810 nm, 109 mW at 10%). The lines represent fits of activation rate *k*_ACT_ to a linear or quadratic function of power *P* for one- or two-photon activation with parameters *b* and *a*, respectively. **e**, Confocal (top) and STED (bottom) images of the same sample following activation by a 405-nm laser. Scale bars: 5 µm (**b**,**c**) and 1 µm (**e**). Source data

To evaluate the compatibility of the PaX photoactivation mechanism with live imaging, we first prepared PaX_560_ constructs (Fig. 3a) containing mitochondria-targeting triphenylphosphonium (**19**) and lysosome-targeting pepstatin A (**20**) moieties, as these selected organelles represent the extreme pH values found within the cell (pH 7.8 for the mitochondrial matrix and pH 4.5 in the lysosomal lumen). COS-7 cells were co-incubated with **19** and MitoTracker Deep Red and imaged with confocal microscopy before and after photoactivation with 355-nm light (Fig. 3b and Supplementary Video 1). The resulting fluorescence of **19-CF** co-localized strongly with the MitoTracker signal (Pearson correlation coefficient *r* = 0.94). Similarly, COS-7 cells concurrently labelled with the pepstatin A conjugate **20** and the lysosome-targeting fluorophore SiR-lysosome^20^ demonstrated colocalization after photoactivation with *r* = 0.84. These results confirmed that the photoactivation mechanism is compatible with live-cell imaging in both high- and low-pH cellular environments.

Self-labelling protein tags, such as HaloTag and SNAP-tag, are well-established tools for targeting synthetic fluorophores to specific proteins in live-cell imaging^47^. Seeking to exploit this targeting strategy, we prepared the HaloTag-specific chloroalkane derivative (**21**) and the SNAP-tag specific *O*^6^-benzylguanine derivative (**22**) of PaX_560_ (Fig. 3a). Chloroalkane derivatives of PaX_480_, PaX_525_ and PaX+_560_ were additionally prepared (Supplementary Figs. 13 and 23–25).

Upon covalent linking of PaX_560_-Halo (**21**) with the HaloTag protein, we observed a 7.8-fold increase in the photoactivation rate of the dye (Supplementary Fig. 14a–d). Complete reaction of **21** with HaloTag with only a slight excess (~1.1 equiv.) of the protein was confirmed by mass spectroscopy (Supplementary Fig. 14b,d). No major fluorescence intensity changes were observed for **21-CF** covalently bound to HaloTag in comparison to free **21-CF** in buffered solution (Supplementary Fig. 14e,f). However, a similar labelling efficiency and a greater fluorogenic response were observed upon binding of PaX_560_–SNAP (**22**) to SNAP-tag, with an 11-fold increase in photoactivation rate (Supplementary Fig. 13a–d) and a 3.3-fold fluorescence intensity increase of SNAP-tag-bound **22-CF** in comparison to free **22-CF** (Supplementary Fig. 15e,f).

We then assessed the feasibility of two-photon activation of PaX_560_–Halo (**21**) with 810-nm near-infrared (NIR) light, as shifting the excitation wavelength from UV to the NIR range reduces phototoxicity and increases imaging depth in tissues. U2OS cells stably expressing a vimentin–HaloTag fusion construct^48^ were labelled with compound **21** and imaged with a confocal microscope equipped with a subpicosecond pulsed laser (Fig. 3c). The activation rate constant was determined for selected areas of the same sample by mono-exponential fitting of the activation rates measured with variable powers of a UV laser for one-photon activation (355 nm) or a subpicosecond pulsed laser for two-photon activation in the NIR (810 nm). Two-photon activation was confirmed by the nearly quadratic (1.84) dependence on the power of the excitation light (Fig. 3d). A pre-activated region of the same sample was further imaged using STED (at 660 nm) to resolve vimentin filaments with subdiffraction resolution, confirming that live-cell STED was readily possible with compound **21** (Fig. 3e). Live-cell STED time-lapse imaging further highlighted the cell dynamics after photoactivation (Supplementary Video 2).

**Fig. 4:**
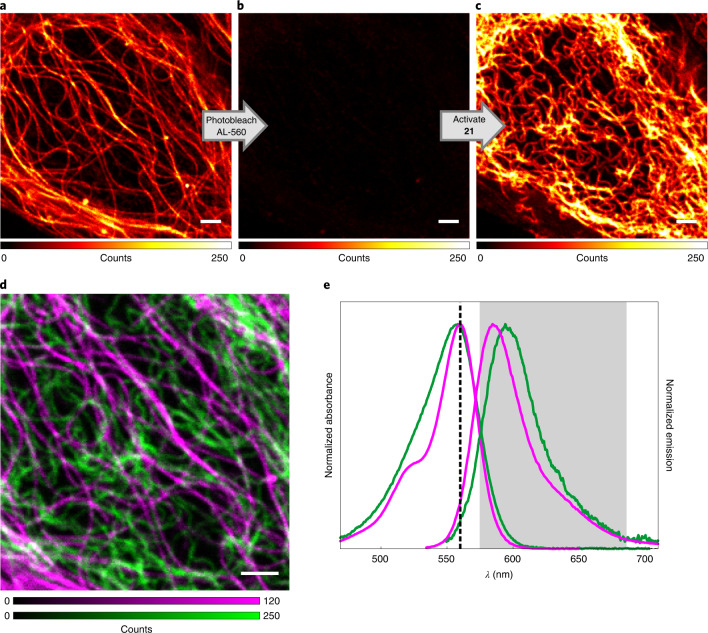
Channel duplexing with PaX labels. **a**–**c**, Confocal imaging of U2OS cells labelled with Abberior LIVE 560 tubulin (AL-560, 500 nM) and vimentin filaments labelled with compound **21** (100 nM) before (**a**) and after (**b**) photobleaching of AL-560 and after photoactivation by a 405-nm laser of **21** (**c**). **d**, Combined pseudo two-colour image showing tubulin (magenta) and vimentin (green) filaments obtained by sequential imaging (**a**–**c**). **e**, Absorption and emission spectra of AL-560 (magenta) and **21-CF** (green), with the excitation laser (dashed line) and detection window (grey) indicated. Scale bars, 2 µm (**a**–**d**). Source data

Photoactivatable fluorophores can also be utilized together with regular ‘always-active‘ fluorescent dyes having similar spectral properties for colour duplexing within a single excitation/detection channel, effectively doubling the number of available imaging channels in a confocal or STED system^12^, provided that bleaching of the ‘always-active’ dye does not result in cell damage. To demonstrate this possibility with PaX labels, U2OS cells stably expressing a vimentin–HaloTag fusion protein were concurrently labelled with PaX_560_–Halo (**21**) and an Abberior LIVE 560 tubulin (AL-560) probe, then imaged by confocal microscopy using a single detection channel (Fig. 4a–e). First, the AL-560-labelled tubulin filaments were visualized (Fig. 4a), followed by AL-560-photobleaching with high-intensity 560-nm excitation light (Fig. 4b). Compound **21** was then, in turn, photo-activated with a 405-nm laser to reveal the **21-CF**-labelled vimentin structure (Fig. 4c).

**Fig. 5:**
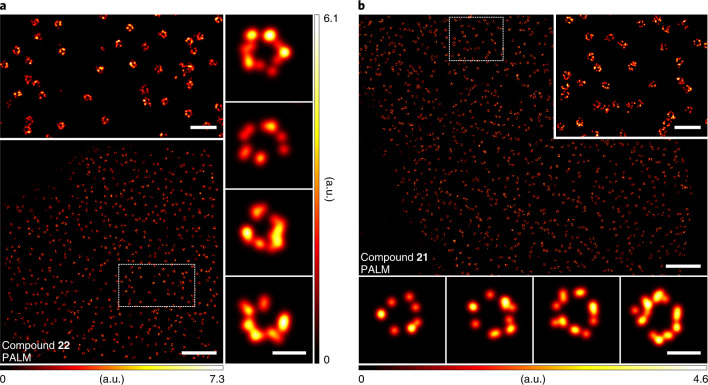
PALM imaging of NPCs in living cells using self-labelling PaX_560_ substrates. **a**, Bottom: PALM image of U2OS stably expressing a NUP107–SNAP-tag construct labelled with **22**. Top: magnified view of the region marked in the overview image. Right column: magnified individual NPCs. **b**, Bottom: PALM image of U2OS cells stably expressing a NUP96-HaloTag construct labelled with **21**. Inset: magnified view of the region marked in the overview image. Bottom row: magnified individual NPCs. Scale bars: 2 μm (**a**,**b**, main), 500 nm (**a**,**b**, top insets), 100 nm (**a**, right column; **b**, bottom row).

We explored the utility of the self-labelling protein tag substrates **21** and **22** for live-cell SMLM. U2OS cells stably expressing a SNAP-tag fusion with NUP107^49^ were labelled with PaX_560_–SNAP (**22**) and imaged with PALM (Fig. 5a). The reconstructed image shows largely complete circumferential labelling, which is remarkable given the one-to-one dye-to-protein ratio, highlighting the efficient labelling and efficient detection of activated PaX. Similarly, U2OS cells stably expressing HaloTag fusion proteins with NUP96^45^ (another NPC protein) were labelled with PaX_560_–Halo (**21**). The reconstructed image (Fig. 5b) resolved the structural elements of the NPCs with even greater efficiency. Fixation of live-labelled samples (with HaloTag and SNAP-tag fusion proteins) also allowed PALM imaging with similar contrast (Supplementary Fig. 16). Thus, the established fixation and permeabilization treatments used to preserve NUP structures^45^ for super-resolution imaging do not affect the performance of PaX labels.

### Multiplexing of PaX labels by selective photoactivation

Given the difference in photoactivation rates for the PaX dyes, we surmised that two complementary labels could be used for multiplexing purposes by sequentially applying a lower and a higher dose of activation light, to first convert one fluorophore (for example, PaX_560_) while preserving the more difficult to activate (for example, PaX_480_) until higher light doses are applied. We tested this first by confocal imaging (Supplementary Fig. 17a–e) and next by two-colour single-detector PALM imaging (Supplementary Fig. 17f) in fixed cells. We further demonstrated sequential activation in live cells (Supplementary Fig 18) by confocal imaging, using the organelle- (PaX_560_–Mito **19** or PaX_560_–Lyso **20**) and HaloTag-specific (PaX_480_–Halo, **23**) labels.

### Utilizing PaX labels in MINFLUX nanoscopy

**Fig. 6:**
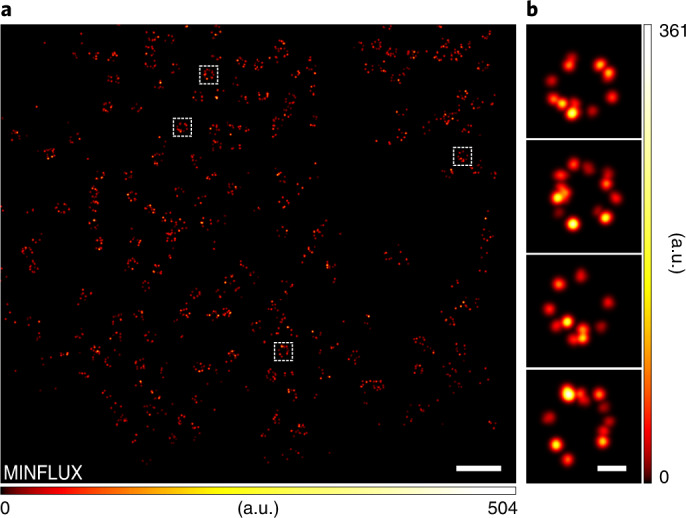
MINFLUX imaging of NPCs using PaX_560_. **a**, MINFLUX image of NPCs in HeLa-Kyoto cells expressing NUP107-mEGFP labelled with anti-GFP nanobodies conjugated to **14**. **b**, Individual NPCs, as marked in **a**. Scale bars: 500 nm (**a**) and 50 nm (**b**).

Finally, we tested the PaX labels in MINFLUX nanoscopy^1,4^, a recent technique that localizes individual fluorophores using an excitation beam with an intensity minimum (zero). Fixed HeLa-Kyoto cells expressing mEGFP fused to NUP107 were labelled with anti-GFP nanobodies bearing **14** and imaged by MINFLUX (Fig. 6a), yielding images of largely complete NPCs (Fig. 6b). On average, molecules were localized 106 times, utilizing 116 photons in the final MINFLUX iteration, and accounting for a mean label precision of 3.7 nm (s.d.).

## Conclusion

We have introduced a general design strategy for caging-group-free, bright- and live-cell-compatible photoactivatable dyes, suitable for a wide range of optical microscopy and nanoscopy techniques, including PALM, STED and MINFLUX. The unique structural feature of these PaX dyes is the combination of a light-responsive 3,6-diaminoxanthone core functionalized with an intramolecular alkene radical trap, to give a highly compact and intrinsically uncharged, intact cell-membrane-permeable label. Under one- or two-photon activation, these compounds rapidly assemble into highly photostable fluorescent pyronine dyes. By changing the substitution pattern of PaX dyes, the photoactivation kinetics as well as the spectral properties can be tuned, allowing for both multiplexed pseudocolour as well as conventional multicolour imaging. The utility and versatility of PaX dyes is illustrated with a diverse range of target-specific probes and labelling strategies, for fixed- and live-cell super-resolution fluorescence microscopy experiments. We expect that our methodology will further stimulate the development of photoactivatable probes and sensors for biological imaging and material science. Further improvements to PaX fluorophores will benefit applications in MINFLUX imaging and the recently proposed MINSTED nanoscopy^50^.

## Methods

Detailed procedures for the synthesis of all compounds and their characterizations, as well as methods sample preparation, live and fixed-cell labelling for microscopy and nanoscopy, are provided in the Supplementary Information. Image acquisition conditions for confocal and STED (Supplementary Table 3) and PALM (Supplementary Table 4), as well as detailed procedures for image processing and rendering, are provided in the Supplementary Information.

### Statistics and reproducibility

All biochemical or spectroscopic data were obtained in triplicate with similar results. All staining/labelling of cells was performed in triplicate. Cells for microscopy were selected at random during the imaging session; sufficient microscopy images were collected, from experience, to ensure their representation of the sample.

### Cell culture

COS-7, HeLa, U2OS-Vim-Halo, U2OS-Vim-SNAP^48,51^ and HK-2xZFN-mEGFP-Nup107^46^ cells were cultured in Dulbecco’s modified Eagle medium (DMEM, 4.5 g l^−1^ glucose) containing GlutaMAX and sodium pyruvate (ThermoFisher 31966), supplemented with 10% (vol/vol) fetal bovine serum (FBS, ThermoFisher 10500064) and 1% Pen Strep (GIBCO, 15140122) in a humidified 5% CO_2_ incubator at 37 °C. Cells were split every 2–4 days or at confluency, and were regularly tested for mycoplasma contamination.

U2OS-ZFN-SNAP-Nup107^49^ and U2OS-NUP96-Halo^45^ cells were cultured in McCoy’s 5a (modified) medium (GIBCO, 26600023) containing l-glutamine and sodium pyruvate, supplemented with 10% (vol/vol) FBS and 1% Pen Strep (GIBCO, 15140122) in a humidified 5% CO_2_ incubator at 37 °C. Cells were split every 2–4 days or at confluency, and were regularly tested to ensure no mycoplasma contamination.

Cell lines with genetically introduced self-labelling tags were verified by confocal microscopy using previously reported fluorophore labels.

### Neuronal culture preparation and labelling

Cultures of dissociated rat hippocampal primary neurons were prepared from postnatal P0-P1 Wistar rats of either sex and cultured on glass coverslips coated with 100 µg ml^−1^ poly-ornithine (Merck KGaA) and 1 µg ml^−1^ laminin (BD Biosciences). Procedures were performed in accordance with the Animal Welfare Act of the Federal Republic of Germany (Tierschutzgesetz der Bundesrepublik Deutschland, TierSchG) and the Animal Welfare Laboratory Animal Regulations (Tierschutzversuchsverordnung). According to the TierSchG and the Tierschutzversuchsverordnung, no ethical approval from the ethics committee is required for the procedure of euthanizing rodents for subsequent extraction of tissues. The procedure for euthanizing P0-P1 rats performed in this study was supervised by animal welfare officers of the Max Planck Institute for Medical Research (MPImF) and conducted and documented according to the guidelines of the TierSchG (permit number assigned by the MPImF: MPI/T-35/18).

Cells were grown in the presence of 1-β-d-arabinofuranosyl-cytosin (Merck KGaA) at 37 °C and 5% CO_2_. Cultures were fixed at 27 days in vitro in 4% paraformaldehyde in PBS, pH 7.4 for 20 min, and quenched for 5 min in PBS supplemented with 100 mM glycine and 100 mM ammonium chloride. Cells were permeabilized for 5 min in 0.1% Triton X-100, blocked with 1% bovine serum albumin for 30 min and incubated with 1 µM **15** diluted in PBS. After extensive washing in PBS, samples were mounted in Mowiol supplemented with DABCO. The identification of axons was facilitated by staining of the axon initial segment with an anti-neurofascin primary antibody (NeuroMab, cat. no. 75-172) and an anti-mouse STAR GREEN (Abberior, cat. no. STGREEN-1001) secondary antibody.

### Reporting summary

Further information on research design is available in the Nature Research Reporting Summary linked to this Article.

## Online content

Any methods, additional references, Nature Research reporting summaries, source data, extended data, supplementary information, acknowledgements, peer review information; details of author contributions and competing interests; and statements of data and code availability are available at 10.1038/s41557-022-00995-0.

## Supplementary information

Supplementary Information

Reporting Summary

Supplementary Video 1 Confocal movie of HeLa cells co-incubated with **19** (200 nM, green) and MitoTracker Deep Red (50 nM, magenta). Conversion to **20-CF** was achieved by a 355-nm laser. Each frame corresponds to ~2.5 s.

Supplementary Video 2 STED movie of vimentin filaments in U2OS cells stably expressing a Vimentin-HaloTag fusion construct labelled with **21** (100 nM). Each frame corresponds to ~14 s.

Supplementary Data Description of custom 405-nm LED light source

## Data Availability

The data supporting the findings of this study are provided within the Paper and its Supplementary Information. The data are also available from the corresponding authors upon reasonable request. Source data are provided with this Paper.

## Source data

Source Data Fig. 1 Statistical source data
Source Data Fig. 3 Statistical source data
Source Data Fig. 4 Statistical source data

## Code availability

The custom code used for image rendering is available at https://github.com/mbossi2015/paper_PaX.
