# Supplementary material for: A general design of caging-group-free photoactivatable fluorophores for live-cell nanoscopy
Source: Nat Chem. 2022 Jul 21;14(9):1013–20. doi: 10.1038/s41557-022-00995-0 (PMC9417988; doi:10.1038/s41557-022-00995-0)
Supplement: Supplementary file 2 — Reporting Summary [file 41557_2022_995_MOESM2_ESM.pdf]

nature portfolio

Corresponding author(s): Dr. Alexey N. Butkevich
Prof. Dr. Stefan W. Hell

Last updated by author(s): Apr 18, 2022

Reporting Summary

Nature Portfolio wishes to improve the reproducibility of the work that we publish. This form provides structure for consistency and transparency in reporting. For further information on Nature Portfolio policies, see our Editorial Policies and the Editorial Policy Checklist.

Statistics

For all statistical analyses, confirm that the following items are present in the figure legend, table legend, main text, or Methods section.

n/a	Confirmed	
☒	☐	The exact sample size (*n*) for each experimental group/condition, given as a discrete number and unit of measurement
☒	☐	A statement on whether measurements were taken from distinct samples or whether the same sample was measured repeatedly
☒	☐	The statistical test(s) used AND whether they are one- or two-sided<br>*Only common tests should be described solely by name; describe more complex techniques in the Methods section.*
☒	☐	A description of all covariates tested
☒	☐	A description of any assumptions or corrections, such as tests of normality and adjustment for multiple comparisons
☒	☐	A full description of the statistical parameters including central tendency (e.g. means) or other basic estimates (e.g. regression coefficient) AND variation (e.g. standard deviation) or associated estimates of uncertainty (e.g. confidence intervals)
☒	☐	For null hypothesis testing, the test statistic (e.g. *F*, *t*, *r*) with confidence intervals, effect sizes, degrees of freedom and *P* value noted<br>*Give P values as exact values whenever suitable.*
☒	☐	For Bayesian analysis, information on the choice of priors and Markov chain Monte Carlo settings
☒	☐	For hierarchical and complex designs, identification of the appropriate level for tests and full reporting of outcomes
☐	☒	Estimates of effect sizes (e.g. Cohen's *d*, Pearson's *r*), indicating how they were calculated

Our web collection on statistics for biologists contains articles on many of the points above.

Software and code

Policy information about availability of computer code

Data collection	Bruker Topspin 3.5, LabSolutions 5.89, Cary Eclipse Scan Application 1.2(147), Cary Eclipse WinUV Scan Application 6.2.0.1588, MatLab R2007a, Imspector (16.1.6905, 16.3.13033, 16.3.13367), LabVIEW 2019 32bit, Andor Solis 4.31.30022, EasyTau 1.4
Data analysis	MestReNova 11.0.3, Imspector (16.1.6905, 16.3.13033, 16.3.13367), ImageJ 1.52i, ImageJ 1.53f51, OriginPro 2020 (64-bit) SR1 9.7.0.188, MatLab R2007a, FluoFit 4.6.6.0

For manuscripts utilizing custom algorithms or software that are central to the research but not yet described in published literature, software must be made available to editors and reviewers. We strongly encourage code deposition in a community repository (e.g. GitHub). See the Nature Portfolio guidelines for submitting code & software for further information.

Data

Policy information about availability of data

All manuscripts must include a data availability statement. This statement should provide the following information, where applicable:

- Accession codes, unique identifiers, or web links for publicly available datasets
- A description of any restrictions on data availability
- For clinical datasets or third party data, please ensure that the statement adheres to our policy

The data supporting the findings of this study are available within the paper and its Supplementary Information and are available from the corresponding author upon reasonable request.

Field-specific reporting

Please select the one below that is the best fit for your research. If you are not sure, read the appropriate sections before making your selection.

☒ Life sciences ☐ Behavioural & social sciences ☐ Ecological, evolutionary & environmental sciences

For a reference copy of the document with all sections, see nature.com/documents/nr-reporting-summary-flat.pdf

Life sciences study design

All studies must disclose on these points even when the disclosure is negative.

Sample size	No sample-size calculation was performed as no biologically-relevant outcome was analyzed. Sufficient microscopy images were collected from experience to ensure their representation of the sample.
Data exclusions	No data were excluded.
Replication	All biochemical or spectroscopic data was performed in triplicate with similar results. All staining/labelling of cells was performed in triplicate.
Randomization	No randomizations were required for the experiments performed. Control of covariates was not relevant to the study as no biologically-relevant outcome was analyzed. Cells for microscopy were selected at random during the imaging session.
Blinding	No blinding was required for the experiments performed as no biologically-relevant outcome was analyzed.

Reporting for specific materials, systems and methods

We require information from authors about some types of materials, experimental systems and methods used in many studies. Here, indicate whether each material, system or method listed is relevant to your study. If you are not sure if a list item applies to your research, read the appropriate section before selecting a response.

Materials & experimental systems

n/a	Involved in the study	
☐	☒	Antibodies
☐	☒	Eukaryotic cell lines
☒	☐	Palaeontology and archaeology
☐	☒	Animals and other organisms
☒	☐	Human research participants
☒	☐	Clinical data
☒	☐	Dual use research of concern

Methods

n/a	Involved in the study	
☒	☐	ChIP-seq
☒	☐	Flow cytometry
☒	☐	MRI-based neuroimaging

Antibodies

Antibodies used	AffiniPure Goat Anti-Rabbit IgG (H+L), Goat, Jackson ImmunoResearch Europe Ltd., 111-005-003<br>AffiniPure Goat Anti-Mouse IgG (H+L), Goat, Jackson ImmunoResearch Europe Ltd., 115-005-003<br>sdAB anti-Mouse kappa light chain (kLC), unconjugated, Camelid, NanoTag Biotechnologies, N1202<br>sdAb anti-Rabbit IgG, unconjugated, Camelid, NanoTag Biotechnologies, N2402<br>FluoTag-X2 anti-GFP unconjugated clone 1H, Camelid, NanoTag Biotechnologies, N0302<br>FluoTag-X2 anti-GFP unconjugated clone 1B, Camelid, NanoTag Biotechnologies, N0303<br>Pan-Neurofascin (extracellular), Mouse, Neuromab, 75-172<br>abberior STAR GREEN, goat anti-mouse IgG, Goat, Abberior, STGREEN-1001<br>Anti-Clathrin heavy chain antibody, Rabbit, Abcam, ab21679<br>Anti-Nup153 antibody [QE5], Mouse, Abcam, ab24700<br>alpha-Tubulin antibody, Mouse, Synaptic Systems, 302 211<br>NUP98 (C39A3) Rabbit mAb, Rabbit, Cell Signalling, #2598
Validation	antibodies and nanobodies were used without further validation as the obtained labelling was clearly compatible with the expected structures. All reagents have already been extensively used by us and others.<br><br>FFluoTag-X2 anti-GFP unconjugated clone 1H, Camelid, NanoTag Biotechnologies, N0302: Manufacturer reports “Recognizes GFP (green fluorescent protein) and common GFP derivatives like EGFP, mEGFP, Sirius, tSapphire, Cerulean, eCFP, mTurquoise, acGFP, Emerald, superecliptic pHluorin, paGFP, superfolder GFP, eYFP, mVenus and Citrine.” utilization in Immunofluorescence (https://nano-tag.com/product/fluotag-x4-anti-gfp/).<br>FluoTag-X2 anti-GFP unconjugated clone 1B, Camelid, NanoTag Biotechnologies, N0303: Manufacturer reports “Recognizes GFP (green fluorescent protein) and common GFP derivatives like EGFP, mEGFP, Sirius, tSapphire, Cerulean, eCFP, mTurquoise, acGFP, Emerald, superecliptic pHluorin, paGFP, superfolder GFP, eYFP, mVenus and Citrine.” utilization in Immunofluorescence (https://

nano-tag.com/product/fluotag-x4-anti-gfp/).
Pan-Neurofascin (extracellular), Mouse, Neuromab, 75-172: Utilized for western blot, immunohistochemistry, immunocytochemistry; reported in 30 references (https://www.labome.com/product/Neuromab/75-172.html).
Anti-Clathrin heavy chain antibody, Rabbit, Abcam, ab21679: Manufacturer reports “confirmed specificity through extensive validation”; utilized in Western blot and Immunocytochemistry; reported in 114 references (https://www.abcam.com/clathrin-heavy-chain-antibody-ab21679.html).
Anti-Nup153 antibody [QE5], Mouse, Abcam, ab24700: Manufacturer reports “ab24700 could also recognise other NPC polypeptides, p250 and p62, apart from Nup153.”; utilization in Immunocytochemistry/Immunofluorescence; reported in 50 references (https://www.abcam.com/nup153-antibody-qe5-ab24700.html).
alpha-Tubulin antibody, Mouse, Synaptic Systems, 302 211: Manufacturer reports “Specific for α-tubulin (glu- and tyr-α-tubulin)”; utilization in Western blot and Immunocytochemistry; reported in 14 references (https://sysy.com/product/302211).
NUP98 (C39A3) Rabbit mAb, Rabbit, Cell Signalling, #2598: Manufacturer reports “NUP98 (C39A3) Rabbit mAb detects endogenous levels of total NUP98 protein”; utilization in Western blot and Immunofluorescence; reported in 37 references (https://www.cellsignal.de/products/primary-antibodies/nup98-c39a3-rabbit-mab/2598).

Eukaryotic cell lines

Policy information about cell lines

Cell line source(s)

COS-7, Hölzl Biotech, AddexBio (T0014002);
HeLa, Kräusslich Group, Virology, University Heidelberg;
U2OS-Vim-Halo, Jakobs Group, Structure & Dynamics of Mitochondria, MPI Multidisciplinary Sciences;
U2OS-Vim-SNAP, Jakobs Group, Structure & Dynamics of Mitochondria, MPI Multidisciplinary Sciences;
HK-2xZFN-mEGFP-Nup107, CLS Cell Lines Service GmbH (300676);
U2OS-ZFN-SNAP-Nup107; CLS Cell Lines Service GmbH (300294);
U2OS-NUP96-Halo, CLS Cell Lines Service GmbH (300448).

Authentication

The cell lines were used without further authentication. Genetically modified cells lines clearly exhibited the expected labeling pattern.

Mycoplasma contamination

Cell lines were regularly tested for mycoplasma contamination and were negative. Primary neuron cultures were not tested.

Commonly misidentified lines
(See ICLAC register)

Not applicable as no commonly misidentified cell lines were used.

Animals and other organisms

Policy information about studies involving animals; ARRIVE guidelines recommended for reporting animal research

Laboratory animals

Postnatal day 0-1 Wistar rats of either sex.

Wild animals

The study did not involve wild animals.

Field-collected samples

The study did not involve samples collected from the field.

Ethics oversight

Procedures were performed in accordance with the Animal Welfare Act of the Federal Republic of Germany (Tierschutzgesetz der Bundesrepublik Deutschland, TierSchG) and the Animal Welfare Laboratory Animal Regulations (Tierschutzversuchsverordnung). According to the TierSchG and the Tierschutzversuchsverordnung no ethical approval from the ethics committee is required for the procedure of sacrificing rodents for subsequent extraction of tissues, as performed in this study. The procedure for sacrificing P0–P2 rats performed in this study was supervised by animal welfare officers of the Max Planck Institute for Medical Research (MPImF) and conducted and documented according to the guidelines of the TierSchG (permit number assigned by the MPImF: MPI/T-35/18).

Note that full information on the approval of the study protocol must also be provided in the manuscript.
