# Supplementary figures and images for: A general design of caging-group-free photoactivatable fluorophores for live-cell nanoscopy
Source: Nat Chem. 2022 Jul 21;14(9):1013–20. doi: 10.1038/s41557-022-00995-0 (PMC9417988; doi:10.1038/s41557-022-00995-0)

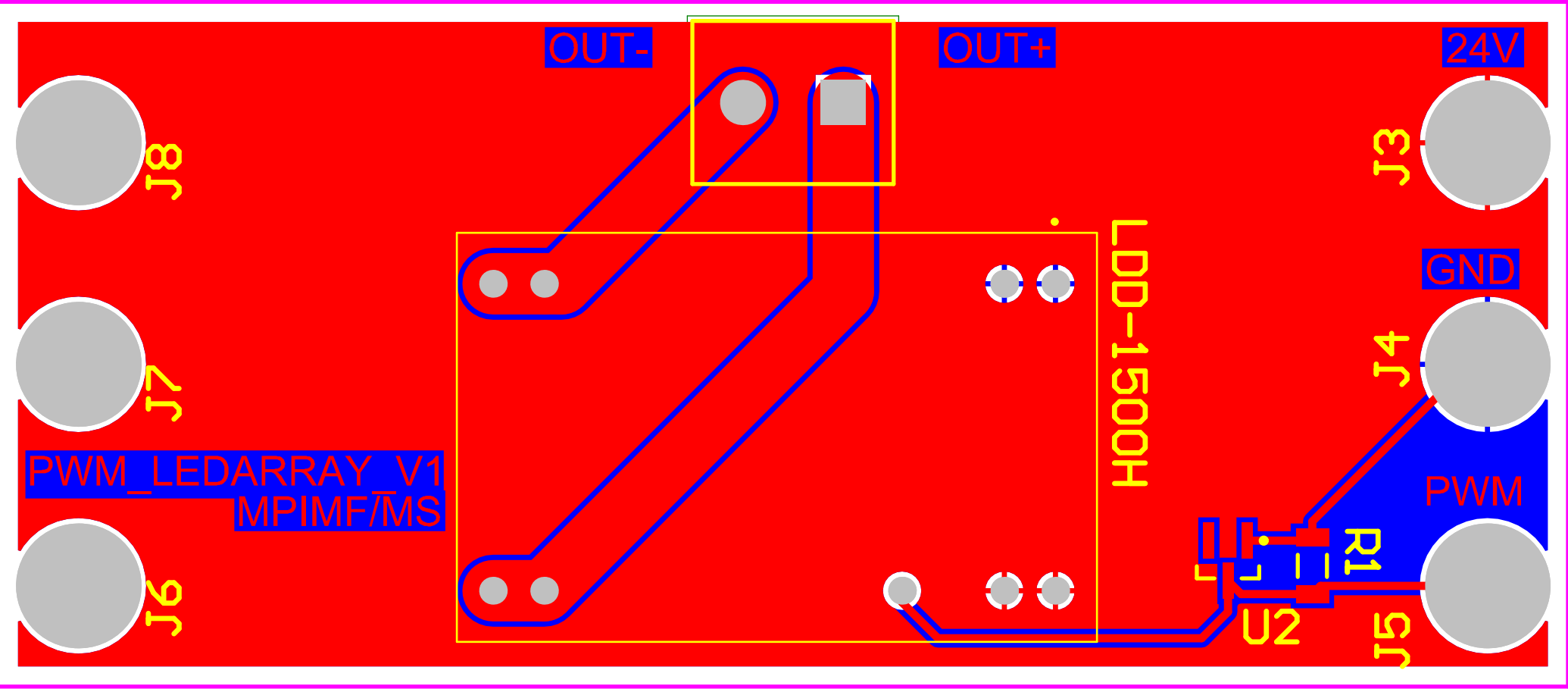

Supplement: Supplementary file 5 — Description of custom 405-nm LED light source [file 41557_2022_995_MOESM5_ESM.zip › custom 405 LED source/electronics/pwm_ledarray_v1_board.pdf]

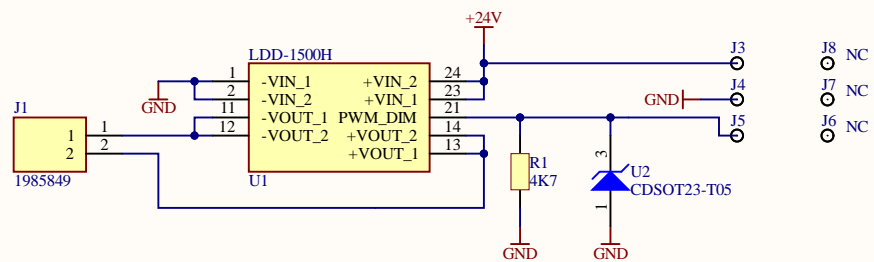

| Title      |                                  |           |
|------------|----------------------------------|-----------|
| Size<br>A4 | Number                           | Revision  |
| Date:      | 8/31/2021                        | Sheet of  |
| File:      | Z:\projects\...\leddriver.SchDoc | Drawn By: |

Supplement: Supplementary file 5 — Description of custom 405-nm LED light source [file 41557_2022_995_MOESM5_ESM.zip › custom 405 LED source/electronics/pwm_ledarray_v1_schematic.pdf]
